# Development and Characterization of Microsatellite Markers Based on the Chloroplast Genome of Tree Peony

**DOI:** 10.3390/genes13091543

**Published:** 2022-08-26

**Authors:** Qi Guo, Lili Guo, Yuying Li, Haijing Yang, Xiaoliang Hu, Chengwei Song, Xiaogai Hou

**Affiliations:** 1 College of Agriculture (College of Tree Peony), Henan University of Science and Technology, Luoyang 471023, China; 2 Luoyang Academy of Agricultural and Forestry Science, Luoyang 471027, China

**Keywords:** tree peony, cpSSRs, polymorphic, diversity, haplotypes

## Abstract

Tree peony (*Paeonia suffruticosa* Andr.) is a famous ornamental and medicinal flowering species. However, few high-efficiency chloroplast microsatellite markers have been developed for it to be employed in taxonomic identifications and evaluation of germplasm resources to date. In the present study, a total of 139 cpSSR loci were identified across eleven tree peony plastomes. Dinucleotide repeat SSRs (97.12%) were most abundantly repeated for the AT motif (58.27%), followed by the TA motif (30.94%) and the TC motif (7.91%). Twenty-one primer pairs were developed, and amplification tests were conducted for nine tree peony individuals. Furthermore, 19 cpSSR markers were amplified on 60 tree peony accessions by a capillary electrophoresis test. Of 19 cpSSR markers, 12 showed polymorphism with different alleles ranging from 1.333 to 3.000. The Shannon’s information index and polymorphism information content values ranged from 0.038 to 0.887 (mean 0.432) and 0.032 to 0.589 (mean 0.268), respectively. The diversity levels for twelve loci ranged from 0.016 (at loci cpSSR-8 and cpSSR-26) to 0.543 (at locus cpSSR-15), averaging 0.268 for all loci. A total of 14 haplotypes (23.33%) were detected in the three populations. The haplotypic richness ranged from 0.949 to 1.751, with a mean of 1.233 per population. The genetic relationship suggested by the neighbor-joining-based dendrogram divided the genotypes into two clusters. The Jiangnan population was allotted to Cluster II, and the other two populations were distributed into both branches. These newly developed cpSSRs can be utilized for future breeding programs, population genetics investigations, unraveling the genetic relationships between related species, and germplasm management.

## 1. Introduction

Tree peony (*P. suffruticosa* Andr.), which belongs to the genus *Paeonia* section *Moutan* DC., is a valuable perennial ornamental and medicinal flowering shrub. China is the origin and center of tree peony cultivation, with 1600 years of planting history. It has been widely cultivated worldwide and contains as many as 2200 cultivars [1,2]. Recently, tree peony has been considered a valuable emerging oil species with rich unsaturated fatty acid and α-linolenic acid contents [3]. However, due to long-term natural selection and domestication processes, the number of cultivated species has been increasing, making it more difficult to study their inheritance and relationship with their origin. To promote more efficient peony breeding, a correct understanding of both is vitally significant for selecting new varieties and arranging and researching the cultivated varieties.

Microsatellite markers, otherwise known as short tandem repeats (STRs) or simple sequence repeats (SSRs), are short units of 1-6 nucleotide tandem repeat sequences widely distributed in eukaryotic and prokaryotic genomes [4,5]. The sequences at both ends of an SSR are often relatively conservative single-copy sequences, which show polymorphism due to the different number or degree of repetitions. Therefore, primers can be designed according to the flanking sequence. The polymorphism primer obtained is based on the genetic parameters (*PIC*: polymorphism information content; *I*: Shannon’s information index). The primer is highly informative when *PIC* > 0.5, reasonably informative when 0.25 < *PIC* < 0.5, and slightly informative when *PIC* < 0.25. Analogously, the level of the Shannon’s information index is similar to the *PIC* value [6]. SSRs with high variability, co-dominance, and extensive genome coverage have been used extensively for characterizing the genetic diversity of tree peony at both the population and species levels to achieve suitable breeding objectives and conservation strategies [5,6,7,8,9,10,11,12].

In previous studies, expressed-sequence-tag-derived simple sequence repeat (EST-SSRs) markers were widely employed to characterize tree peony populations for genetic linkage maps [13], population structure [8,14], and association analysis [15,16,17]. In addition, gene-derived simple sequence repeats (genomic SSRs) were also developed and applied to the genetic diversity of tree peony [11]. Because the chloroplast genome (cpDNA) is a semi-autonomous inheritance, chloroplast microsatellite markers (cpSSRs)—with their simple structure, single-parent inheritance, and independent evolution [18,19]—have the advantages of SSR markers and also the characteristics of the cpDNA. In comparison with EST-SSRs and genomic SSRs, cpSSRs were widely used to study genetic diversity, phylogeography, mating patterns, and genetic conservation. That was due to: (1) the small genome size of cpDNA, (2) the absence of sexual recombination, (3) a relatively high rate of sequence evolution, and (4) amplified homologous regions across related taxa [20,21,22,23,24,25].

However, few cpSSRs have been developed for tree peony germplasms until now, and among them, only six cpSSR loci were developed and selected with a polymorphic basis of 76 *P. delavayi* individuals [26]. As a result, this study developed a group marker based on the chloroplast sequence of five cultivars and six species and analyzed the distribution characteristics of cpSSRs to lay a foundation for developing a tree peony transferable chloroplast. Furthermore, the genetic diversity of 60 peony varieties from three regions was explored for their cytoplasmic genetic differences. The cpSSR markers developed in this study could be used for molecular classification among *Paeonia* to facilitate their identification for conservation.

## 2. Materials and Methods

### 2.1. Chloroplast Sequencing Acquisition

In this study, the chloroplast genome information of eleven peony samples was obtained from the study of Guo et al. [27], which contained 6 species and 5 cultivars. It was used for the development of SSR markers, including *Paeonia lutea* (HMD, GenBank ID: MK701989), *Paeonia jishanensis* (JS, GenBank ID: MK701988), *Paeonia qiui* (LY, GenBank ID: MK701992), *Paeonia potanini* (XY, GenBank ID: MK701991), *Paeonia ostia* (YS, GenBank ID: MK701990), *Paeonia rockii* (ZB, GenBank ID: MF488719), *P*. *suffruticosa* ‘Dou Lv’ (DL, GenBank ID: MK701994), *Paeonia ostii* ‘Feng Dan’ (FD, GenBank ID: MG585274), *P. suffruticosa* ‘Luo Yang Hong’ (LYH, GenBank ID: MK701995), *P. suffruticosa* ‘High Noon’ (ZW, GenBank ID: MK701997), and *P. suffruticosa* ‘Shou An Hong’ (SAH, GenBank ID: MK701996).

### 2.2. Sample Collection and DNA Extraction

**Figure 1:**
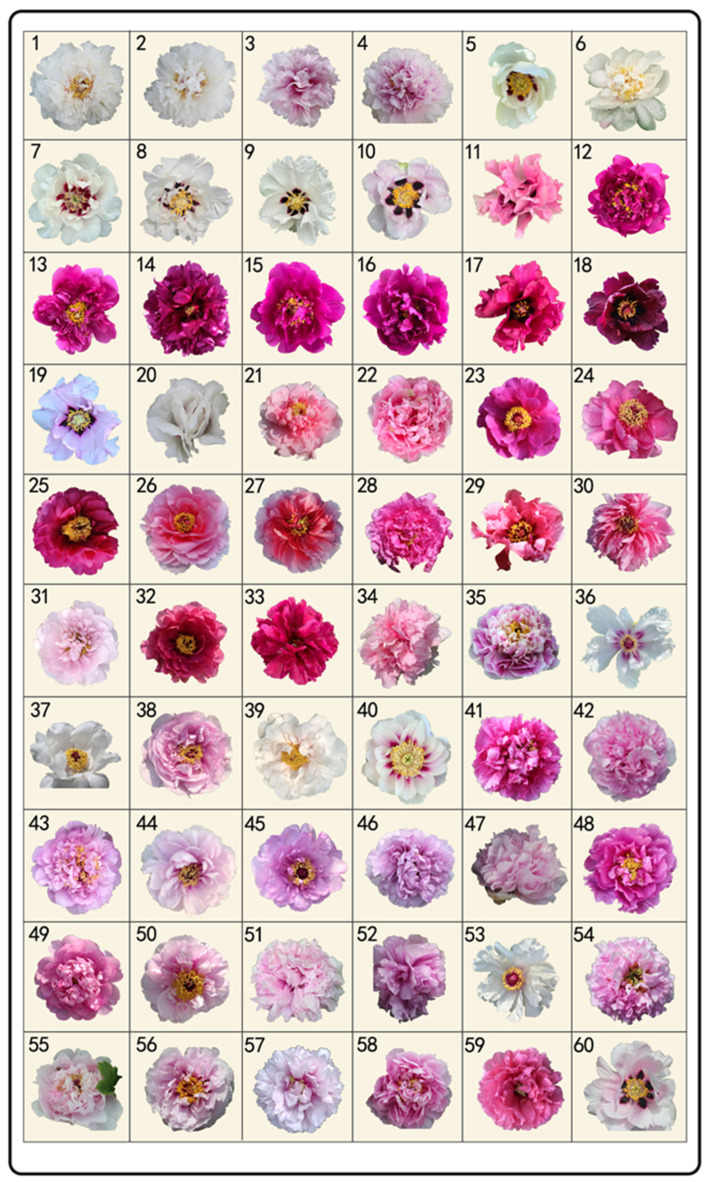
Flower photos showing the sixty tree peony cultivars used in this study.

Sixty tree peony samples from different sources with varying floral colors and types were used to evaluate the gene polymorphism by using designed primers (Figure 1, Table S1). These samples were collected from the Luoyang Academy of Agriculture and Forestry Sciences. Leaflets from all the samples were frozen in liquid nitrogen immediately after their collection in the field and stored in a −80 °C freezer prior to DNA extraction.

The total genomic DNA was extracted using the Super Plant Genomic DNA Kit (Polysaccharides & Polyphenolics-rich) (Tiangen, Beijing, China). DNA quality was assessed by electrophoresis on a 1% agarose gel, and its concentration was measured by a NanoDrop 2000 UV-Vis Spectrophotometer (Thermo Fisher Scientific, Wilmington, DE, USA).

### 2.3. cpSSR Design and Polymorphism Screening

Excavated and developed chloroplast simple sequence repeat (cpSSR) elements were used by GMATA ver. 2.2 software (Chinese Academy of Sciences, Kunming, China) [28] for the eleven tree peony samples using the constraints of more than 5 repeats and a motif size ranging from 2 to 6 bp. After obtaining results from the preliminary screening, SSR loci were selected, and primer pairs were designed. The following constraints were applied: (1) minimum and maximum product sizes of 150–400 bp; (2) annealing temperatures of about 60 °C; (3) a flanking region size ≤2000 bp; (4) deletion of repeat sequence primers and default values for the other parameters.

### 2.4. cpSSR Polymorphisms Screening and Validation

In this study, the fluorescent-labeled M13 primers were used to mark the polymorphism. Briefly, an M13-forward primer with the sequence 5′-TGTAAAACGACGGCCAGT-3′ was used in combination with an M13 primer of the same sequence, labeled at the 5′ end of each forward primer using four fluorescent dyes (FAM, HEX, ROX and TAMRA) [29]. The PCR amplification was performed with a total of 10 μL, containing 2 μL DNA (20 ng/μL), 5 μL 2 × Taq Plus Master Mix II (Dye Plus) (Vazyme Biotech Co., Ltd., Nanjing, China), 1.6 μL dd H_2_O, and 0.6 μL of M13 primer (10 µM) (Ruibio BioTech Co., Ltd., Beijing, China) [30]. The forward and reverse primers were 0.2 μL (10 μM) and 0.6 μL (10 μM), respectively. A BIO-RAD T100 thermal cycler was used for denaturation with 4 min at 94 °C; then 30 cycles at 94 °C for 40 s, 60 °C for 30 s, and 72 °C for 45 s, followed by another 20 cycles at 94 °C for 40 s, 53 °C for 30 s, and 72 °C for 45 s, followed by a final extension of 10 min at 72 °C.

Agarose gel and capillary electrophoresis were used to evaluate the polymorphisms and validate the cpSSR markers for the PCR products. Genomic DNA from nine tree peony cultivars (*P. suffruticosa* ‘Huang He’, *P. suffruticosa* ‘Cai Die Fei Wu’, *P. suffruticosa* ‘Lan Hai Bi Bo’, *P. suffruticosa* ‘Wan Die Yun Feng’, *P. suffruticosa* ‘Gui Fu Ren’, *P. suffruticosa* ‘Ling Hua Zhan Lu’, *P. suffruticosa* ‘Fen Lian’, *P. suffruticosa* ‘Bai Yan Wei’ and *P. suffruticosa* ‘Yan Luo Fen He’) with different colors, floral patterns, and origins were mixed at equal concentrations and volume. The amplified products were separately detected by 1% agarose gel electrophoresis, and the primers with the polymorphism were selected according to those results. After that, sixty tree peony cultivars were used to validate the selected cpSSR markers with capillary electrophoresis technology. The DNA fragments of each sample were resolved with an ABI 3730XL DNA Sequencer (Applied Biosystems, Foster City, CA, USA). The GeneMarker ver. 2.2.0 software (SoftGenetics LLC, State College, PA, USA) was used to confirm the cpSSR loci.

### 2.5. Data Analysis

GenAlEx ver. 6.501 (Australian National University, Canberra ACT 0200, Australia) [31,32] software was used to evaluate the population’s genetic diversity parameters, including the number of different alleles (*Na*), the effective number of alleles (*Ne*), Shannon’s information index (*I*), and diversity (*H*). The polymorphism information content (*PIC*) was calculated using PowerMarker ver. 3.25 software (North Carolina State University, North Carolina, USA) [33]. The number of haplotypes (*A*), number of private haplotypes (*P*), effective number of haplotypes (*Ne*), haplotypic richness (*Rh*), and genetic diversity (*He*) were used in the Haplotype Analysis ver. 1.05 software (Georg-August University, Goettingen, Germany) [34]. Subsequently, the network of cpSSR data was converted to binary data via DataTrans ver. 2003 and neighbor joining (NJ) was constructed and displayed using the R package ‘ggtree’ (University of Hong Kong, Hong Kong SAR, China) (http://www.bioconductor.org/packages/ggtree accessed on 20 December 2021) [35].

## 3. Results and Discussion

**Figure 2:**
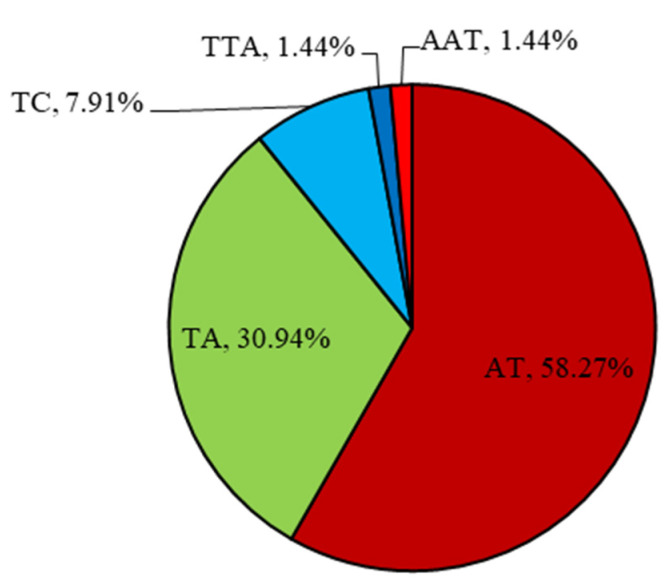
Type and proportion of motif repetition for cpSSRs for eleven different samples of *P. suffruticosa*.

**Figure 3:**
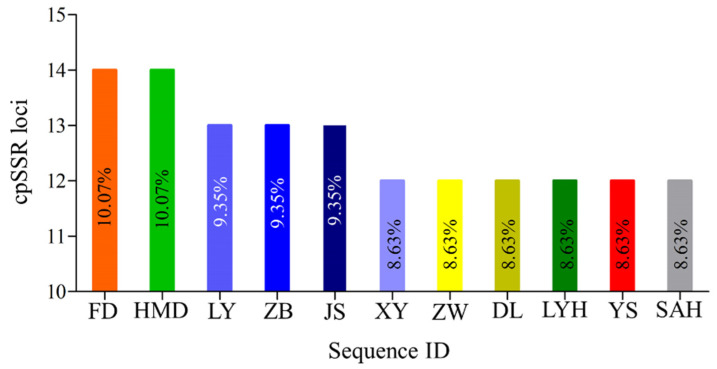
The proportion of cpSSR loci in eleven different samples of *P. suffruticosa* chloroplast genome sequence. Note: FD: *Paeonia ostii* ‘Feng Dan’, HMD: *Paeonia lutea*; LY: *Paeonia qiui*; ZB: *Paeonia rockii*; JS: *Paeonia jishanensis*, XY: *Paeonia potanini*, ZW: *P. suffruticosa* ‘High Noon’, DL: *P. suffruticosa* ‘Dou Lv’, LYH: *P. suffruticosa* ‘Luo Yang Hong’; YS: *Paeonia ostia*, SAH: *P. suffruticosa* ‘Shou An Hong’.

**Figure 4:**
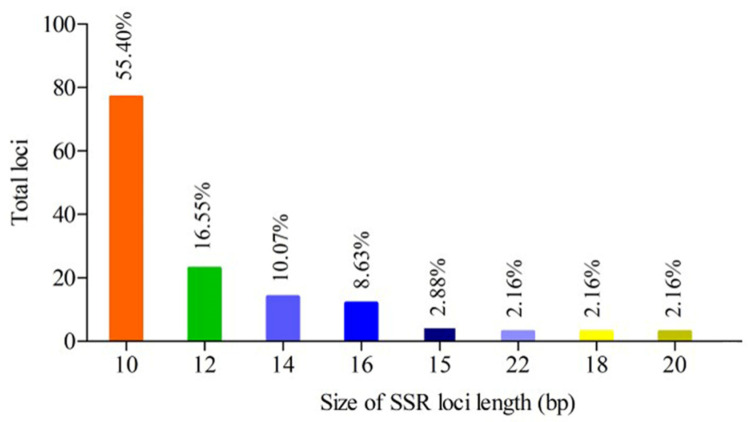
Number and percentage of size of cpSSR loci length for eleven different samples of *P. suffruticosa*.

A total of 139 primer pairs were identified from the chloroplast genome of the eleven samples. The di-nucleotides (135; 97.12%) and tri-nucleotides (4; 2.88%) were motif types. Among them, the AT motif (58.27%) was most abundantly repeated, followed by the TA (30.94%) motif, then the TC motif (7.91%), and the TTA and AAT motifs equally (Figure 2). The proportion of cpSSR loci in the chloroplast genome sequences could be divided into three groups. The first group with the highest cpSSR loci proportion contained two cultivated species (FD and HMD), and the third group with the lowest contained six cultivated species (XY, ZW, DL, LYH, YS, and SAH) (Figure 3). Eight types of cpSSR-length loci were found among the 139 primer pairs, 10 bp being the largest with 77 primer pairs, followed by 12 bp with 23, 14 bp with 14, 16 bp with 12, 15 bp with 4, and 22 bp, 18 bp, and 20 bp with 3 each (Figure 4).

Twenty-one cpSSR markers were obtained by deleting duplicate primer sequences (Table 1); the two primers (cpSSR-16 and cpSSR-21) yielded a weak amplification of nine samples. Ultimately, 19 unique cpSSR markers were acquired with the agarose gel electrophoresis screening test. After that, 19 cpSSR markers were amplified on 60 tree peony accessions with a capillary electrophoresis test. The proportion of primers obtained by two screening tests was 90.48%, indicating that the primer design method and strategy are reasonable. Moreover, this is a common standard and an efficient method for verifying the versatility and the effectiveness of the developed chloroplast markers with initial screening and re-screening tests. For instance, Huang et al. [36] obtained 26 cpSSR primer pairs using these two screening methods to evaluate the genetic diversity of Cupressaceae species.

Among nineteen cpSSR markers, we deleted seven cpSSR markers (cpSSR-2, cpSSR-3, cpSSR-7, cpSSR-10, cpSSR-12, cpSSR-13, and cpSSR-23) with monomorphism, and the other twelve markers with polymorphism were used for the following analysis. The number of alleles (*Na*) ranged from 1.333 (for cpSSR-8, cpSSR-25, and cpSSR-26) to 3.000 (for cpSSR-4) with an average of 2.028. The effective number of alleles (*Ne*) per marker ranged from 1.017 to 2.422, averaging 1.508 for all the loci. Shannon′s information index (*I*) ranged from 0.038 (at loci cpSSR-8 and cpSSR-26) to 0.887 (at locus cpSSR-5), with a mean of 0.432 per marker. The diversity (*H*) ranged from 0.016 (at loci cpSSR-8 and cpSSR-26) to 0.543 (at locus cpSSR-15). The *PIC* ranged from 0.032 (at loci cpSSR-8, cpSSR-19, cpSSR-25, and cpSSR-26) to 0.589 (at locus cpSSR-5), with a mean of 0.268 per marker (Table 2). This was lower than in 13 populations (*PIC* = 0.386) of 76 *P. delavayi* individuals [26].

A total of 14 haplotypes (23.33%) were detected in the three populations, and their number per population ranged from 2 to 8. The effective number of haplotypes (*N_eh_*) ranged from 1.707 to 3.687, with an average of 2.464 per population. The haplotypic richness (*R_h_*) ranged from 0.949 to 1.751, with a mean of 1.233 per population. The gene diversity (*He*) levels for twelve loci ranged from 0.442 to 0.669 (Table 3). The results showed that the high genetic diversity of the Zhongyuan population might be a result of its sample size being the largest. Eleven haplotypes were identified based on 12 cpSSR markers among 60 tree peony samples and they all had the private haplotypes (Table 4). Of these, there were eight private haplotypes (haplo-3, haplo-4, haplo-5, haplo-6, haplo-7, haplo-8, haplo-9, and haplo-10). There were four rare haplotypes (haplo-4, haplo-6, haplo-7, and haplo-9), and the frequency value was below 5% (Table S2).

Compared with the development, evaluation, and application of EST-SSR [6,7,9,11,37] or Genomic-SSR [38] markers in tree peony, there has been little related research of cpSSR markers [39] until now. The *PIC* and *I* ranged from 0.36 to 0.85 (mean = 0.69) and 0.83 to 2.14 (mean = 1.56), respectively (based on 30 EST-SSR markers from 36 accessions) [37]. The *PIC* and *I* ranged from 0.58 to 0.85 (mean = 0.73) and 0.9973 to 2.0745 (mean = 1.5494), respectively (based on 25 EST-SSR from 31 accessions). The *Ne* ranged from 1.4 to 4.7 with an average of 2.4 pre-locus; *PIC* varied from 0.257 to 0.794 (average = 0.468) (based on 12 Genomic-SSR markers from 48 accessions) [38]. In this study, the values of *PIC* and *I* were less diverse and lower than the other two types of genetic markers. Comparable results have been observed in other studies using different SSR markers in *Quercus infectoria* [40] and *Pinus sylvestris* L. [20]. Yang et al. [41] developed 70 cpSSR loci and tested them for amplification success and polymorphism in 16 samples; after that, 10 loci were used to estimate a total of 83 individuals from three populations in China. The effective number of haplotypes ranged from 4.55 to 13.36, and the haplotypic richness ranged from 8.04 to 16.00. Gene diversity ranged from 0.81 to 0.97 (average = 0.89). The results showed that the employment of higher haplotypic richness and genetic diversity than those used in our study might allow for greater amplification efficiency.

**Figure 5:**
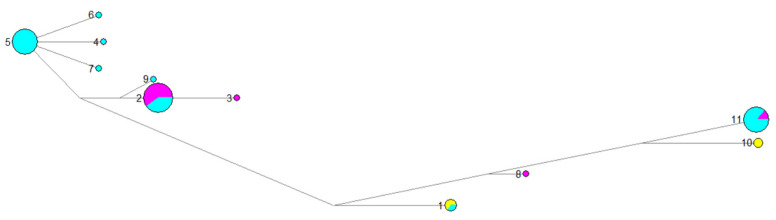
Distribution of 11 chloroplast haplotypes detected in *P. suffruticosa.* Note: 1-11: haplotype 1-11; yellow color: Jiangnan group; purple color: Xibei group; blue color: Zhongyuan group.

In the haplotype spectrum diagram (Figure 5), haplotypes 3, 4, 6, 7, 8, and 9 contained one sample. Haplotype 2 contained the most samples, including twelve and eight, respectively, in the Xibei and Zhongyuan populations. In total, the Zhongyuan population contained the largest number of haplotypes, perhaps because the Zhongyuan population had the largest sample size.

**Figure 6:**
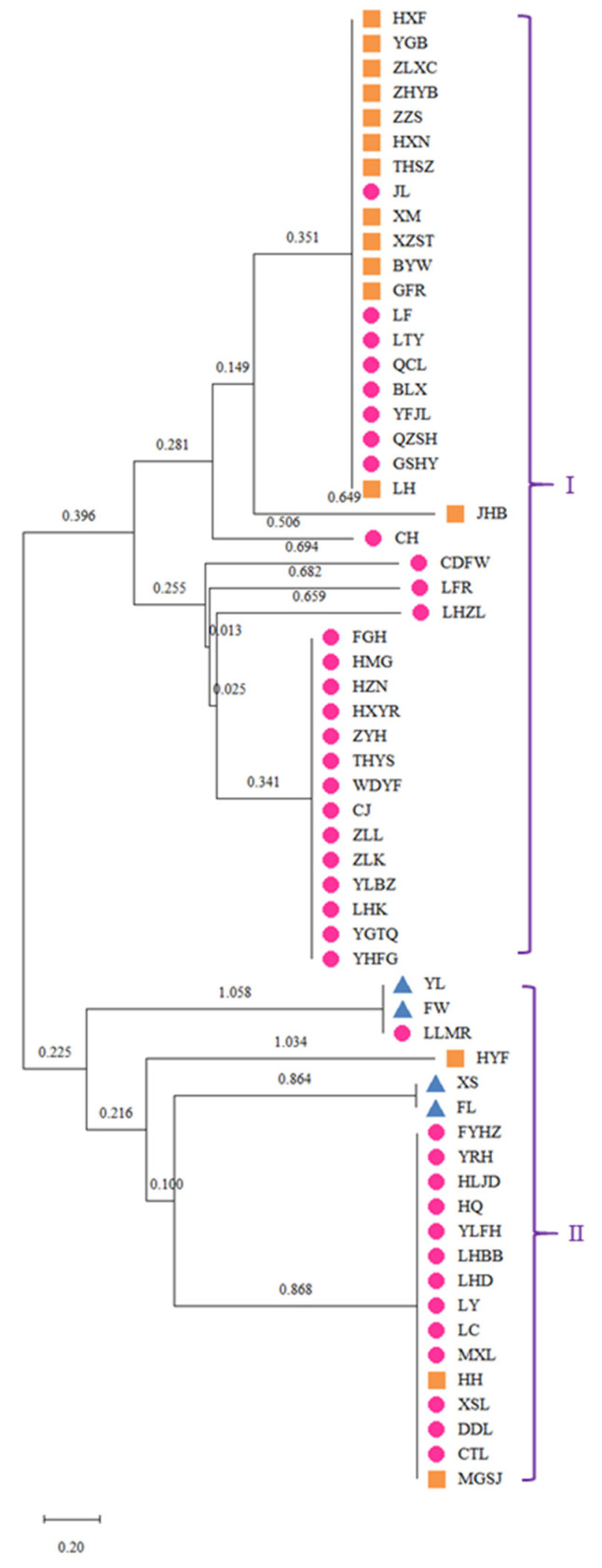
Neighbor-joining (NJ) dendrogram of 60 tree peony cultivars derived from diversities of all 12 polymorphic cpSSR markers. Note: orange color with a square: Xibei group; pink color with a circle: Zhongyuan group; blue color with a triangle: Jiangnan group.

Organelle DNA markers are more reliable for taxonomic identification and classification than nuclear DNA markers [42,43]. With NJ cluster analysis, chloroplast microsatellite markers could have revealed the genetic relationships of the present study’s individuals. The NJ dendrogram generated for the 60 tree peony cultivars grouped all the cultivars into two clusters (Figure 6). The Zhongyuan population was distributed in both clusters; Cluster I contained about twice as many individuals as Cluster II. Cluster I comprised 39 genotypes, which contained a larger number of the Xibei population (13, 81.25%). Cluster II comprised 21 genotypes, which contained the entire Jiangnan population. The four cultivars from Jiangnan were divided into two subgroups with two varieties per subgroup and bootstrap values of 1.058 and 0.864, respectively. The colors of YL and FW are white and those of XS and FL are pink. The NJ tree for these individuals had similar SRAP markers to those of 20 tree peony cultivars [44], and similar SSR markers to 48 tree peony accessions [38]. This result about the origin of the cultivated peony varieties is complex and diverse. In spite of cpSSR markers having the advantages of chloroplast genomes in origin and evolution studies, more accurate results need to be obtained, which might discover the sequences of cpSSR markers that amplified results and were mined by base sequence analysis.

In summary, the group of cpSSR primer pairs were designed and can therefore be used to evaluate genetic diversity, population structure, and phytogeographical patterns. The development, synthesis, and verification of cpSSR markers using chloroplast genome information about tree peony are also reported. Using this set of cpSSR markers, additional research can be implemented to the association maps for this species.

## Figures and Tables

**Table 1:** Characteristics of twenty-one cpSSR markers for tree peony.

No.	MarkerID	Primer Left Sequence	Primer Right Sequence	Tm	Product Size	Motif
1	cpSSR-1	ATGAAAGGCGTCCATTGTCT	CGGAAGCCCTTTTGAAAGAA	60	344	(TA)_7_
2	cpSSR-2	ATTTGGCTCTCACGCTCAAT	CGCGGCATAGTACTCTTTCT	58	399	(AT)_5_
3	cpSSR-3	GTAGGACTCCGGCCATAACA	AGGGGTTGTGGATACTGCTG	59	363	(AT)_5_
4	cpSSR-4	CAAGACCGGAGCTATCAACC	TCGTGGAGGGATTTTACCAA	59	253	(TA)_7_
5	cpSSR-5	ATCAGCATTCTCCCCTCAAA	TCAAACAGCAAAGAGCTGGA	59	392	(AT)_6_
6	cpSSR-6	CGGACCGGACTCTCTTGTAT	TCCGATAGCTGGCTTTTCTC	59	227	(AT)_5_
7	cpSSR-7	TCGGTTGGCTCATACGAAAT	TGCCTGAAAGTTGGATAGGG	60	288	(TA)_5_
8	cpSSR-8	GTTTGCTGTGGTCCCTGTCT	CCATAGAAAGACAAGCCCCTTT	60	253	(AT)_8_
9	cpSSR-9	TTACCGCCCGTTCACTTAAT	CCGACATAGACCGAAAAGGA	59	362	(AT)_5_
10	cpSSR-10	CCCAACCCTACCCATTTCTT	GGACTTCCGGTTCAGACATC	59	198	(AT)_5_
11	cpSSR-11	AGCTGAAGCGGGCTCTTACT	AACCCAAGATGCCAGACTTTT	60	299	(TC)_5_
12	cpSSR-12	AGTTCCAGAGCTCGTCCAAA	TAAATTCCGCGGTCGAACTA	60	339	(TA)_6_
13	cpSSR-13	ATTGCACACAATCGGAATCA	CCCAATTGATGGGAGAGAGA	59	217	(AT)_5_
14	cpSSR-15	TCCAGCTCTTTGCTGTTTGA	CACGGGTTCAAATCCTGTCT	59	163	(AT)_5_
15	cpSSR-16	TCGGTAACGCGACATAAAGA	CCCTTTTCCAAGGGTTATGG	59	312	(AT)_5_
16	cpSSR-17	GCTGTGGTCCCTGTCTCAGT	CCATTCCAACCAAACTAAGCTC	60	400	(AT)_8_
17	cpSSR-19	CACCTGCCATTACGTCATCA	TTGAACAACAAAGGGCGATT	60	378	(AAT)_5_
18	cpSSR-21	CAAGCAAGATTCATCGACTGG	CTCGATTCGTGGGTCAAAAC	60	400	(AT)_10_
19	cpSSR-23	AGCTCCTCGCGAATGATAAA	AACAAACCGGGGACAATTC	59	370	(TTA)_5_
20	cpSSR-25	TGTGGTCCATGTCTCAGTTCA	TCCATTCCAACCAAACTAAGC	59	400	(AT)_9_
21	cpSSR-26	CAACCCCACCCATTTCTTTT	GGTGTGAACCTTTCGGGATA	60	303	(AT)_5_

**Table 2:** Genetic diversity indicators for the 12 chloroplast SSR markers.

Marker	*Na*	*Ne*	*I*	*H*	*PIC*
cpSSR-1	2.000	1.622	0.568	0.381	0.324
cpSSR-4	3.000	1.892	0.667	0.381	0.472
cpSSR-5	2.667	2.422	0.887	0.543	0.589
cpSSR-6	2.000	1.728	0.603	0.414	0.351
cpSSR-8	1.333	1.017	0.038	0.016	0.032
cpSSR-9	2.000	1.104	0.189	0.090	0.121
cpSSR-11	2.667	1.784	0.709	0.430	0.403
cpSSR-15	2.333	1.670	0.620	0.396	0.367
cpSSR-17	1.667	1.142	0.209	0.116	0.164
cpSSR-19	2.000	1.641	0.574	0.387	0.332
cpSSR-25	1.333	1.051	0.086	0.044	0.032
cpSSR-26	1.333	1.017	0.038	0.016	0.032
Mean	2.028	1.508	0.432	0.268	0.268

*Na*: number of alleles; *Ne*: effective number of alleles; *I*: Shannon′s information index; *H*: diversity; *PIC*: polymorphism information content.

**Table 3:** Haplotype diversity in three tree peony populations based on 12 cpSSR markers.

Population	*N*	*A*	*N_eh_*	*R_h_*	*He*	*D^2^_sh_*
Jiangnan	4	2	2.000	1.000	0.667	150.222
Xibei	16	4	1.707	0.949	0.442	142.522
Zhongyuan	40	8	3.687	1.751	0.747	195.178
Mean	-	4.667	2.464	1.233	0.619	162.641

*N*: sample size in each population; *A*: number of haplotypes detected in each population; *N_eh_*: effective number of haplotypes; *R_h_*: haplotypic richness; *He*: genetic diversity; *D^2^_sh_*: mean genetic distance between individuals.

**Table 4:** The identified haplotypes and their frequencies based on 12 cpSSR markers among 60 tree peony samples.

Haplotype Code	cpSSR-1	cpSSR-4	cpSSR-5	cpSSR-6	cpSSR-8	cpSSR-9	cpSSR-11	cpSSR-15	cpSSR-17	cpSSR-19	cpSSR-25	cpSSR-26	Frequencies
**haplo-1**	362	269	404	263	271	384	315	149	418	398	418	319	0.05
**haplo-2**	362	271	413	245	271	386	313	149	420	398	418	319	0.33
**haplo-3**	362	271	413	245	271	388	313	149	420	398	418	319	0.02
**haplo-4**	362	273	410	245	271	386	313	149	420	398	418	283	0.02
**haplo-5**	362	273	410	245	271	386	313	149	420	398	418	319	0.23
**haplo-6**	362	273	410	245	271	386	313	149	420	398	420	319	0.02
**haplo-7**	362	273	410	245	283	386	313	149	420	398	418	319	0.02
**haplo-8**	362	277	404	263	271	386	315	179	418	392	418	319	0.02
**haplo-9**	362	279	413	245	271	386	313	149	420	398	418	319	0.02
**haplo-10**	364	273	404	263	271	386	313	185	418	392	418	319	0.03
**haplo-11**	364	273	404	263	271	386	318	179	420	392	418	319	0.25

## Supplementary Materials

The following supporting information can be downloaded at: https://www.mdpi.com/article/10.3390/genes13091543/s1, Table S1: Information on 60 tree peony cultivars of genetic diversity analysis using the cpSSR primers developed based on chloroplast sequences, Table S2: The identified private haplotypes and their frequencies among 11 haplotypes.
