# Supplementary material for: Development and Characterization of Microsatellite Markers Based on the Chloroplast Genome of Tree Peony
Source: Genes (Basel). 2022 Aug 26;13(9):1543. doi: 10.3390/genes13091543 (PMC9498374; doi:10.3390/genes13091543)
Supplement: Supplementary file 1 [file genes-13-01543-s001.zip › genes-1804004-supplementary.pdf]

Table S1. Information on 60 tree peony cultivars of genetic diversity analysis using the cpSSR primers developed based on chloroplast sequences

No.	Accession	Abbreviation	Flower shape	Flower colour	Bloom time	Group
1	*Paeonia suffruticosa cv.* 'Yu Lou'	YL	Chrysanthemum flowered	White	Normal	Jiangnan
2	*Paeonia suffruticosa cv.* 'Feng Wei'	FW	Lotus Form	White	Early	Jiangnan
3	*Paeonia suffruticosa cv.* 'Xi Shi'	XS	Chrysanthemum flowered	Pink	Early	Jiangnan
4	*Paeonia suffruticosa cv.* 'Fei Lian'	FL	Rose shaped	Pink	Normal	Jiangnan
5	*Paeonia suffruticosa cv.* 'Huang He'	HH	Single Dahlia	Yellow	Normal	Xibei
6	*Paeonia suffruticosa cv.* 'Ju Hua Bai'	JHB	Amenone Form	White	Normal	Xibei
7	*Paeonia suffruticosa cv.* 'Gui Fu Ren'	GFR	Chrysanthemum flowered	White	Normal	Xibei
8	*Paeonia suffruticosa cv.* 'Bai Yan Wei'	BYW	Amenone Form	White	Normal	Xibei
9	*Paeonia suffruticosa cv.* 'Xue Zhong Song Tan'	XZST	Chrysanthemum flowered	White	Normal	Xibei
10	*Paeonia suffruticosa cv.* 'Xiong Mao'	XM	Single Dahlia	White	Normal	Xibei
11	*Paeonia suffruticosa cv.* 'Hong Xian Nv'	HXN	Crown Form	Pink	Early	Xibei
12	*Paeonia suffruticosa cv.* 'Hong Yang Fei'	HYF	Single Dahlia	Red	Normal	Xibei
13	*Paeonia suffruticosa cv.* 'Mei Gui Sa Jin'	MGSJ	Crown Form	Purple	Normal	Xibei
14	*Paeonia suffruticosa cv.* 'Zi Zhu Sha'	ZZS	Crown Form	Red	Early	Xibei
15	*Paeonia suffruticosa cv.* 'Zi Hai Yin Bo'	ZHYB	Amenone Form	Purple	Normal	Xibei
16	*Paeonia suffruticosa cv.* 'Zi Lou Xiang Cu'	ZLXC	Crown Form	Purple	Normal	Xibei
17	*Paeonia suffruticosa cv.* 'Hei Xuan Feng'	HXF	Single Dahlia	Black	Early	Xibei
18	*Paeonia suffruticosa cv.* 'Ye Guang Bei'	YGB	Single Dahlia	Black	Early	Xibei
19	*Paeonia suffruticosa cv.* 'Lan He'	LH	Single Dahlia	Blue	Normal	Xibei
20	*Paeonia suffruticosa cv.* 'Tao Hua San Zhuan'	THSZ	Crown Form	Blue	Normal	Xibei
21	*Paeonia suffruticosa cv.* 'Fu Gui Hong'	FGH	Crown Form	Red	Normal	Zhongyuan
22	*Paeonia suffruticosa cv.* 'Fei Yan Hong Zhuang'	FYHZ	Pavilion type/Rose shaped	Red	Normal	Zhongyuan
23	*Paeonia suffruticosa cv.* 'Hong Mei Gui'	HMG	Rose shaped	Purple-red	Normal	Zhongyuan
24	*Paeonia suffruticosa cv.* 'Hong Zhu Nv'	HZN	Crown Form	Red	Normal	Zhongyuan

25	*Paeonia suffruticosa cv*. 'Hong Xia Ying Ri'	HXYR	Rose shaped/Pavilion type	Purple-red	Late	Zhongyuan
26	*Paeonia suffruticosa cv*. 'Guan Shi Hong Yu'	GSHY	Rose shaped	Red	Normal	Zhongyuan
27	*Paeonia suffruticosa cv*. 'Ying Ri Hong'	YRH	Pavilion type	Red	Normal	Zhongyuan
28	*Paeonia suffruticosa cv*. 'Zhao Yang Hong'	ZYH	Rose shaped/Chrysanthemum flowered	Red	Normal	Zhongyuan
29	*Paeonia suffruticosa cv*. 'Huo Lian Jin Dan'	HLJD	Lotus Form/Crown Form	Red	Early	Zhongyuan
30	*Paeonia suffruticosa cv*. 'Hong Qiao'	HQ	Chrysanthemum flowered	Red	Normal	Zhongyuan
31	*Paeonia suffruticosa cv*. 'Tao Hua Yu Shuang'	THYS	Pavilion type	Pink	Late	Zhongyuan
32	*Paeonia suffruticosa cv*. 'Chen Hui'	CH	Chrysanthemum flowered	Red	Normal	Zhongyuan
33	*Paeonia suffruticosa cv*. 'Qi Zhuang Shan He'	QZSH	Crown Form	Purple-red	Normal	Zhongyuan
34	*Paeonia suffruticosa cv*. 'Yin Fen Jin Lin'	YFJL	Crown Form	Pink	Late	Zhongyuan
35	*Paeonia suffruticosa cv*. 'Wan Die Yun Feng'	WDYF	Crown Form	Multicolored	Normal	Zhongyuan
36	*Paeonia suffruticosa cv*. 'Cai Die Fei Wu'	CDFW	Lotus Form	Pink	Normal	Zhongyuan
37	*Paeonia suffruticosa cv*. 'Lou Lan Mei Ren'	LLMR	Crown Form	Pink	Late	Zhongyuan
38	*Paeonia suffruticosa cv*. 'Yan Luo Fen He'	YLFH	Globular Form	Blue	Late	Zhongyuan
39	*Paeonia suffruticosa cv*. 'Bai Lian Xiang'	BLX	Lotus Form/Crown Form	White	Early	Zhongyuan
40	*Paeonia suffruticosa cv*. 'Cai Ju'	CJ	Chrysanthemum flowered	Pink	Early	Zhongyuan
41	*Paeonia suffruticosa cv*. 'Lan Hai Bi Bo'	LHBB	Golden-Circle Form	Blue	Early	Zhongyuan
42	*Paeonia suffruticosa cv*. Zi Luo Lan'	ZLL	Crown Form	Pink	Early	Zhongyuan
43	*Paeonia suffruticosa cv*. 'Zi Lan Kui'	ZLK	Crown Form	Purple	Early	Zhongyuan
44	*Paeonia suffruticosa cv*. 'Lan Hu Die'	LHD	Single Dahlia	Blue	Early	Zhongyuan
45	*Paeonia suffruticosa cv*. 'Lan Yu'	LY	Rose_shaped/Pavilion type	Blue	Early	Zhongyuan
46	*Paeonia suffruticosa cv*. 'Lan Fu Rong'	LFR	Pavilion type	Blue	Normal	Zhongyuan
47	*Paeonia suffruticosa cv*. 'Chi Lan'	CL	Globular Form	Blue	Late	Zhongyuan
48	*Paeonia suffruticosa cv*. 'Mang Xing Lan'	MXL	Amenone Form/Crown Form	Blue	Normal	Zhongyuan
49	*Paeonia suffruticosa cv*. 'Yin Lin Bi Zhu'	YLBZ	Crown Form	Purple	Normal	Zhongyuan
50	*Paeonia suffruticosa cv*. 'Lan Hua Kui'	LHK	Crown Form/Amenone Form	Blue	Normal	Zhongyuan

51	*Paeonia suffruticosa cv*. 'Xi Shi Lan'	XSL	Crown Form	Blue	Late	Zhongyuan
52	*Paeonia suffruticosa cv*. 'Da Duo Lan'	DDL	Crown Form	Blue	Late	Zhongyuan
53	*Paeonia suffruticosa cv*. 'Yu Guo Tian Qing'	YGTQ	Crown Form	Blue	Normal	Zhongyuan
54	*Paeonia suffruticosa cv*. 'Qing Cui Lan'	QCL	Crown Form	Blue	Normal	Zhongyuan
55	*Paeonia suffruticosa cv*. 'Lan Tian Yu'	LTY	Crown Form	Blue	Normal	Zhongyuan
56	*Paeonia suffruticosa cv*. 'Yu Hou Feng Guang'	YHFG	Rose_shaped	Blue	Normal	Zhongyuan
57	*Paeonia suffruticosa cv*. 'Chui Tou Lan'	CTL	Crown Form	Blue	Late	Zhongyuan
58	*Paeonia suffruticosa cv*. 'Ling Hua Zhan Lu'	LHZL	Pavilion type	Purple	Late	Zhongyuan
59	*Paeonia suffruticosa cv*. 'Lu Fen'	LF	Crown Form	Pink	Normal	Zhongyuan
60	*Paeonia suffruticosa cv*. 'Jia Li'	JL	Chrysanthemum flowered	White	Normal	Zhongyuan

Table S2. The identified private haplotypes and their frequencies among 11 haplotypes

Haplotype Code	cpSSR-1	cpSSR-4	cpSSR-5	cpSSR-6	cpSSR-8	cpSSR-9	cpSSR-11	cpSSR-15	cpSSR-17	cpSSR-19	cpSSR-25	cpSSR-26	Frequencies
**haplo-3**	362	271	413	245	271	388	313	149	420	398	418	319	0.06
**haplo-4**	362	273	410	245	271	386	313	149	420	398	418	283	0.03
**haplo-5**	362	273	410	245	271	386	313	149	420	398	418	319	0.35
**haplo-6**	362	273	410	245	271	386	313	149	420	398	420	319	0.03
**haplo-7**	362	273	410	245	283	386	313	149	420	398	418	319	0.03
**haplo-8**	362	277	404	263	271	386	315	179	418	392	418	319	0.06
**haplo-9**	362	279	413	245	271	386	313	149	420	398	418	319	0.03
**haplo-10**	364	273	404	263	271	386	313	185	418	392	418	319	0.50
